# Feasibility of *TP53*-Mutated ctDNA Monitoring in High-Grade Endometrial Cancer Using Routine NGS

**DOI:** 10.3390/cancers18071102

**Published:** 2026-03-28

**Authors:** Regine Marlin, Mehdi Jean-Laurent, Clarisse Joachim, Alexis Vallard, Sabrina Pennont, Valerie Suez-Panama, Mickaelle Rose, Sylviane Ulric-Gervaise, Sylvie Lusbec, Odile Bera, Aude Aline-Fardin, Coralie Ebring

**Affiliations:** 1 Pôle de Biologie, Génétique des Cancers, CHU de Martinique, Fort-de-France, 97200 Martinique, France; 2 Pole MFME, Chirurgie Gynécologique et Mammaire, CHU de Martinique, Fort-de-France, 97200 Martinique, France; mehdi.jean-laurent@chu-martinique.fr (M.J.-L.); coralie.ebring@gmail.com (C.E.); 3 Pôle de Cancérologie Hématologie Urologie, Registre Général des Cancers de la Martinique, CHU de Martinique, Fort-de-France, 97200 Martinique, France; 4 Pôle de Cancérologie Hématologie Urologie, CHU de Martinique, Fort-de-France, 97200 Martinique, France; 5 Pôle de Biologie, Centre de Ressources Biologiques, CHU de Martinique, Fort-de-France, 97200 Martinique, France; 6 Plateforme Régionale d’Oncologie de Martinique (GIP PROM), CHU de Martinique, Fort-de-France, 97200 Martinique, France; 7 Pôle de Biologie, Anatomopathologie, CHU de Martinique, Fort-de-France, 97200 Martinique, France

**Keywords:** ctDNA monitoring, *TP53* mutation, high-grade endometrial cancer, recurrence detection, minimal residual disease, next-generation sequencing, liquid biopsy

## Abstract

Background/Objectives: High-grade endometrial cancer (EC) is associated with poor outcomes, particularly in populations with a high burden of aggressive histologies. There is a critical need for accessible biomarkers to improve prognostic assessment and guide clinical management. Methods: In this study, we evaluated the feasibility and clinical relevance of monitoring circulating tumor DNA (ctDNA) by tracking somatic *TP53* mutations using a routine next-generation sequencing (NGS) assay already implemented in diagnostic practice. Results: Among 21 patients with high-grade EC carrying *TP53* mutations in the primary tumor, ctDNA was detectable in over 75% during follow-up. Baseline ctDNA detection strongly correlated with advanced disease: none of the FIGO I tumors were ctDNA-positive at diagnosis, whereas 73% of FIGO > I tumors showed detectable ctDNA. Patients with ctDNA detected at baseline had significantly poorer outcomes, with a 2-year recurrence-free survival (RFS) of 18% versus 60% and a 2-year overall survival (OS) of 40% versus 78%. Longitudinal monitoring revealed that postoperative persistence or reappearance of ctDNA was consistently associated with disease progression, often preceding radiological relapse. Conversely, early ctDNA clearance (at M4–M8) was associated with more favorable clinical trajectories. Conclusions: These findings highlight the potential role of ctDNA as a real-time molecular marker of minimal residual disease and tumor dynamics. Our results demonstrate that *TP53*-based ctDNA tracking using a standard NGS panel is feasible, sensitive, and clinically informative in high-grade EC. This approach may contribute to improving prognostic stratification and enabling more personalized, responsive clinical management, particularly in high-risk populations.

## Simple Summary

High-grade endometrial cancer is an aggressive disease with a poor prognosis, particularly when diagnosed at an advanced stage. Blood-based biomarkers able to monitor tumor evolution could help improve patient management. In this study, we evaluated whether tumor DNA circulating in the blood could be used to monitor disease progression in patients with high-grade endometrial cancer. By targeting mutations of the *TP53* gene, which is frequently altered in these tumors, we followed patients before and after surgery using a routine sequencing test. We found that the presence or reappearance of circulating tumor DNA was strongly associated with cancer recurrence and poor outcomes, whereas its disappearance after treatment was linked to better prognosis. These results suggest that circulating tumor DNA could become a useful, non-invasive tool to monitor treatment response and anticipate relapse.

## 1. Introduction

High-grade endometrial carcinoma, which is the most frequent gynecological cancer, remains a major challenge due to its aggressive behavior and its often late diagnosis [1]. The recent integration of molecular classification into clinical practice has profoundly transformed the management of endometrial cancer (EC). It now allows the identification of four subgroups with distinct oncogenic and prognostic profiles—*POLE*-ultramutated, MSI/hypermutated, copy-number low/microsatellite stable, and serous-like/copy-number high—thus guiding therapeutic strategies more closely aligned with the underlying biological mechanisms [2,3,4,5,6]. Pathogenic *POLE* mutations are now recognized as being associated with an excellent prognosis, and no benefit from adjuvant therapy has been demonstrated in this subtype, even in high-grade tumors [7,8,9]. Conversely, MSI/dMMR tumors, in advanced or recurrent settings, may benefit from immunomodulatory therapies, including immune checkpoint inhibitors, which offer new therapeutic opportunities [10,11,12]. For women with high-risk EC harboring somatic p53 abnormalities, the results of the PORTEC-3 trial, incorporated into the 2025 recommendations, confirm the superiority of adjuvant chemoradiotherapy compared with radiotherapy alone [12,13].

Despite these advances, prognosis remains poor for many patients, particularly in Martinique, where there is an overrepresentation of non-endometrioid subtypes [14], known for their aggressiveness [15]. These tumors have recently been associated with *CCNE1* amplification [16], an alteration characteristic of replication stress, a major mechanism contributing to the poor prognosis of high-grade forms [17]. In this context, it is essential to develop simple and accessible tools to monitor the unfavorable evolution of these tumors and to adapt clinical management.

Circulating tumor DNA (ctDNA) has recently emerged as a promising biomarker, both prognostic and predictive, in several cancers [18,19]. The strong concordance between mutations identified in the primary tumor and those detected in plasma [20,21] highlights its value as a non-invasive tool for assessing tumor burden and molecular evolution [18,21,22]. In lung cancer, ctDNA is now essential for detecting *EGFR* mutations, including the T790M resistance variant, which guides targeted therapy when tumor tissue is not available [23,24]. Similarly, in breast cancer, the recent SERENA-6 trial demonstrated that serial ctDNA monitoring can detect *ESR1* resistance mutations months before radiological progression, enabling earlier therapeutic intervention [25,26]. In operable patients, postoperative ctDNA detection is also a strong prognostic marker, associated with higher recurrence risk and reduced survival [27]. More recently, the interest of ctDNA in endometrial tumors has been explored. Several studies suggest that its preoperative detection is associated with high-grade tumors and with unfavorable prognosis [28,29,30,31,32]. When targeting somatic alterations identified through next-generation sequencing (NGS), ctDNA detection appears more frequent in tumors diagnosed at an advanced stage, with high tumor volume, or with serous-like histology [30].

Given the high proportion of aggressive EC observed in Martinique and their unfavorable evolution, we evaluated the relevance of using ctDNA as a surveillance tool by targeting the *TP53* mutation identified in each patient’s primary tumor. To ensure immediate transposability into clinical practice, we used a commercial NGS panel already integrated into the laboratory’s diagnostic workflow. The aim of this study was to determine whether ctDNA detection could serve as an accessible prognostic marker, capable of reflecting the evolution of high-grade tumors and contributing to a more individualized patient management.

## 2. Methods

### 2.1. Study Design and Subjects

All patients with high-grade EC diagnosed between November 2019 and March 2021 in Martinique were included in the study which was approved by the “Committee for the Protection of Individuals Sud Mediterranee IV” (ID-RCB:2018-A209154). Histological classification was performed by a dedicated pathologist according to the World Health Organization recommendations [33]. All patients underwent an initial assessment including computed tomography and bone scintigraphy. Patients were enrolled at the time of surgery, which predominantly involved hysterectomy with bilateral salpingo-oophorectomy. For patients who did not undergo surgery, the inclusion date corresponded to the date of biopsy or endometrial curettage. Detailed clinical data were collected, including age at diagnosis, histological type, TNM stage, FIGO stage, BMI, parity, menopausal status, and comorbidities (arterial hypertension, diabetes, etc.). Disease stage was defined according to the FIGO 2009 staging system, which was the reference classification at the time of patient inclusion. Follow-up visits were scheduled every four months at 4, 8, 12, 16, 20, and 24 months after inclusion and were primarily designed for biological sampling for ctDNA analysis. During each follow-up visit, patients underwent a clinical and gynecological examination. Radiological assessments were not performed according to a study-specific protocol but followed standard recommendations and routine clinical practice in force at the time of patient management. Imaging was performed when clinically indicated, mainly using contrast-enhanced computed tomography (CT), to assess disease recurrence or progression. Disease progression or recurrence was defined by radiological evidence of new lesions or progression of existing disease, in conjunction with clinical findings. At inclusion, biological samples included a frozen tumor specimen when available, a peripheral blood sample, and Cell-Free DNA Collection Tubes (Roche) for baseline ctDNA assessment. Human samples were processed by the “Centre de Ressources Biologiques de Martinique”. Written informed consent for genetic analyses was obtained from all patients.

### 2.2. Tumor Molecular Analysis

All patients benefited from molecular characterization of the primary tumor at diagnosis. When frozen tissue was available, whole-exome sequencing (WES) was performed as part of a dedicated study aimed at identifying the oncogenic mechanisms driving high-grade tumor development [34]. When only FFPE material was available, a targeted NGS gene panel routinely used in the laboratory—including *TP53*—was applied. This dual approach ensured systematic identification of somatic mutations, including *TP53*, enabling subsequent ctDNA tracking. MSI was assessed by amplifying the Pentaplex mononucleotide repeat panel (BAT-25, BAT-26, NR-21, NR-22, and NR-24). Fragment analysis was performed on an ABI PRISM 3500 genetic analyzer (Applied Biosystems^®^, Foster City, CA, USA), as previously described [35]. From extracted tumor DNA, MSI (dMMR) status was defined by the instability of at least two microsatellite markers. MSI results were systematically compared with mismatch repair (MMR) protein expression assessed by immunohistochemistry. Following surgery, patients received adjuvant treatment (chemotherapy followed by radiotherapy, chemotherapy alone, radiotherapy, or brachytherapy), in accordance with recommendations for high-grade EC [35,36].

### 2.3. ctDNA Follow-Up

Only tumors harboring *TP53* mutations were eligible for ctDNA monitoring. Plasma ctDNA was extracted at baseline (M0) and during follow-up (M4, M8, M12, M16, M20, M24) using the Maxwell RSC platform (Promega Corporation, Madison, WI, USA). ctDNA was analyzed using the mini-Homologous Recombination Solution panel (SOPHiA GENETICS SA, Saint-Sulpice, Switzerland), a compact four-gene panel routinely used in the diagnostic laboratory. This assay incorporates molecular barcoding, enabling detection of very low-frequency variants. Libraries were sequenced on a MiSeq instrument (Illumina, San Diego, CA, USA), and bioinformatic processing was performed using SOPHiA DDM, Rolle, Switzerland. Mutations identified in the primary tumor were genotyped directly in plasma and considered detected when supported by at least three independent reads and a minimum allele frequency (MAF) of 0.01. This threshold is consistent with the analytical performance and error profile of the Illumina MiSeq platform and with previously published ctDNA studies using non-UMI amplicon-based NGS [37,38].

### 2.4. Statistical Analysis

The primary endpoint was recurrence-free survival (RFS), defined as the interval between diagnosis and first relapse or death. The secondary endpoint was overall survival (OS), defined as time from diagnosis to death from any cause. Kaplan-Meier survival analysis was performed, and subgroup differences were evaluated using the log-rank test. A *p*-value < 0.05 was considered statistically significant. Disease stage was categorized as early (FIGO I) or advanced (FIGO > II). ctDNA detection at baseline (M0) was analyzed as a clinical variable.

## 3. Result

### 3.1. Clinicopathologic Characteristics

A total of 21 patients with high-grade EC were included in the final analysis. The cohort consisted of 11 uterine papillary serous carcinomas (UPSC), 8 uterine carcinosarcomas (UCS), and 3 mixed tumors. The median age at diagnosis was 73 years (range 62–88).

No pathogenic *POLE* mutations were identified in the cohort, and all tumors were microsatellite stable (MSS/pMMR). Most tumors belonged to the copy-number–altered molecular subgroup and were frequently associated with *CCNE1* amplification, as previously reported [34].

**Figure 1:**
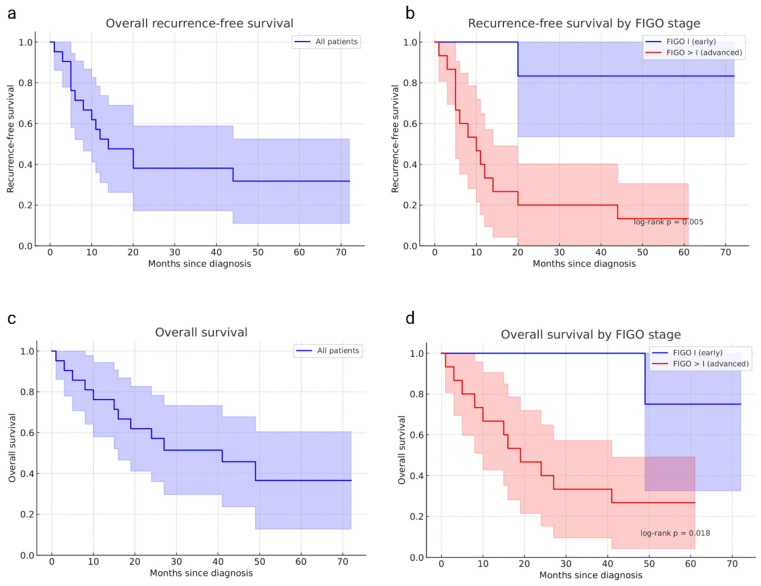
Longitudinal ctDNA monitoring and survival outcomes in high-grade endometrial cancer. (**a**) Kaplan–Meier curve of recurrence-free survival (RFS) for the entire cohort (blue), with 95% confidence intervals represented by shaded areas. (**b**) Kaplan–Meier analysis of RFS stratified by FIGO stage, showing significantly poorer outcomes for advanced-stage disease (FIGO > I, red) compared with early-stage disease (FIGO I, blue). Shaded areas indicate 95% confidence intervals. (**c**) Kaplan–Meier curve of overall survival (OS) for the entire cohort (blue), with corresponding 95% confidence intervals. (**d**) Kaplan–Meier analysis of OS stratified by FIGO stage, demonstrating reduced survival in advanced-stage tumors (red) compared with FIGO I disease (blue). The *p*-values displayed on panels (**b**) and (**d**) were obtained using the log-rank test.

At 3 years, the recurrence-free survival (RFS) and overall survival (OS) rates for the entire cohort were 38% (95% CI, 17–59) and 51% (95% CI, 30–73), respectively (Figure 1). A total of 71% of patients were diagnosed at an advanced FIGO stage (>I), and these patients exhibited markedly poorer outcomes. The 2-year RFS and OS rates in advanced-stage tumors were 20% (95% CI, 0–40) and 40% (95% CI, 15–65), compared with 83% (95% CI, 54–100) and 100% in FIGO I tumors, respectively. The difference in RFS between early and advanced stages was statistically significant (log-rank *p* ≈ 0.005; Figure 1b).

**Figure 2:**
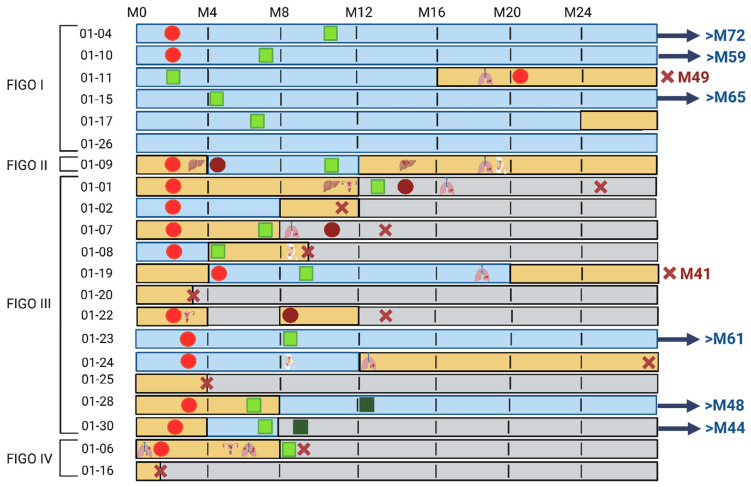
Swimmer plot illustrating individual patient trajectories, including timing of treatments, ctDNA positivity, radiologic progression, and survival status. Each row represents one patient, grouped according to FIGO stage at diagnosis. Surgery is not depicted, as it corresponds to the baseline timepoint (M0). Patients ID06 and ID16 did not undergo surgery due to poor clinical condition. First-line chemotherapy is indicated by red circles, and second-line chemotherapy by dark red (burgundy) circles. Radiotherapy or brachytherapy is represented by light green square and dark green square, respectively. When available, treatments administered after disease progression are indicated in the swimmer plot; two patients received additional chemotherapy after progression. Recurrences are listed according to the organ involved. Deaths are marked with a red cross. Bar color indicates ctDNA status: blue for ctDNA-negative, yellow for ctDNA-positive, and grey when no sample was available. Arrows at the end of a bar, followed by “ >M72”, indicate that the patient was still alive at that time point.

Only one of the six patients diagnosed at FIGO stage I experienced disease recurrence, occurring at 16 months after surgery. Among the remaining five patients, three showed no evidence of recurrence with long-term follow-up (59, 65, and 72 months). Two patients were lost to follow-up after the 24-month evaluation, including one patient in whom ctDNA was detected at month 24 in the absence of documented clinical progression (Figure 2). In contrast, most patients diagnosed at FIGO stage > I experienced relapse or clinical deterioration, with a median time to recurrence of 10 months. The most frequent metastatic sites were the lung (33%), peritoneum (20%), liver (9%), and bone (4.5%), consistent with recurrence patterns typically observed in high-grade endometrial carcinomas (Figure 2).

According to ESGO/ESTRO/ESP and NCCN guidelines, most patients underwent surgery. Only two patients (IDs 06 and 16) were deemed ineligible due to poor performance status. Eight patients received adjuvant chemoradiotherapy, two received radiotherapy alone, and five received chemotherapy alone, the latter mostly due to early recurrence preventing completion of radiotherapy (Figure 2).

### 3.2. ctDNA Follow-Up

#### 3.2.1. Baseline ctDNA Detection Was Strongly Associated with Disease Stage

**Figure 3:**
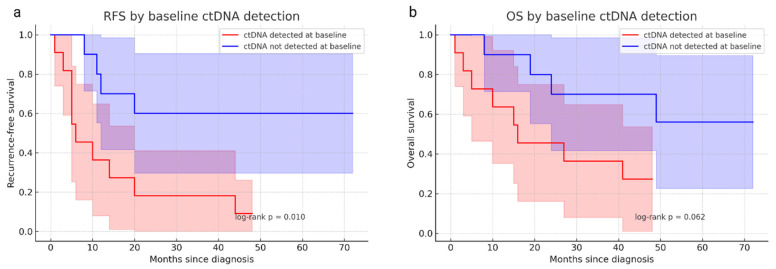
Patient survival data according to baseline ctDNA detection. Kaplan–Meier survival curves comparing patients with detectable ctDNA at baseline (red) and those without detectable ctDNA (blue). (**a**) Recurrence-free survival (RFS) with 95% confidence intervals shaded. Patients with baseline ctDNA detection exhibited significantly poorer RFS (log-rank *p* = 0.010). (**b**) Overall survival (OS) with 95% confidence intervals shaded. A trend toward reduced OS was observed in ctDNA-positive patients (log-rank *p* = 0.062). ctDNA: circulating tumor DNA.

Baseline ctDNA detection–defined as the blood sample collected at surgery–was strongly associated with disease stage. None of the FIGO I tumors showed detectable ctDNA at M0, whereas 67% (10/15) of FIGO > I tumors were ctDNA-positive at baseline, with allele fractions ranging from 0.01 to 0.3 (Figure 2). These findings are summarized in Table 1, which details ctDNA detection patterns across disease stages. Baseline ctDNA positivity was strongly associated with poorer outcomes: patients with detectable ctDNA at M0 had significantly reduced recurrence-free survival (2-year RFS: 18% vs 60%, log-rank *p* = 0.010) and showed a trend toward inferior overall survival (2-year OS: 40% vs 78%, log-rank *p* = 0.062). Figure 3 illustrates these survival differences, showing Kaplan–Meier curves for RFS and OS according to baseline ctDNA detection.

Interestingly, four advanced-stage tumors (IDs 02, 08, 23, 24) remained ctDNA-negative at M0 (Figure 2). In two of these cases, the absence of detection coincided with limited tumor invasion or isolated peritoneal disease.

#### 3.2.2. Post-Operative ctDNA Status Reflects Therapeutic Response

Among the 11 patients with baseline ctDNA positivity (all FIGO > I), ctDNA kinetics closely reflected the biological response to therapy. Three patients showed ctDNA clearance at M4 following first-line adjuvant chemotherapy, consistent with an initial biological response. Among these patients, one remains alive at last follow-up, while two died at 41 and 44 months, respectively (Figure 2). Four patients showed persistent ctDNA at M4 despite first-line adjuvant chemotherapy, three of whom died rapidly between M8 and M24, indicating early resistance to systemic treatment. The fourth patient experienced delayed ctDNA clearance at M8 after radiotherapy, suggesting a biological response to locoregional treatment; however, this patient subsequently died at M48. Among the remaining four patients with baseline ctDNA positivity, three died very rapidly before the M4 evaluation, precluding longitudinal assessment of ctDNA kinetics, and one patient, for whom no plasma sample was available at M4, showed persistent ctDNA at M8 and died at M12. Overall, these findings indicate that early ctDNA clearance is associated with treatment response, whereas ctDNA persistence reflects primary therapeutic resistance.

#### 3.2.3. Post-Operative ctDNA Status Was Correlated with Disease Progression

Post-operative ctDNA detection showed a strong association with clinical progression. In most patients, the re-emergence or persistence of ctDNA during follow-up coincided with the appearance of new distant lesions or with clinical deterioration (Figure 2). Among patients with detectable ctDNA at baseline, ctDNA became undetectable after surgery in several cases but reappeared before or at the time of radiologic progression. For example, patient 09 showed ctDNA clearance at M4 and M8 in parallel with chemotherapy response, followed by ctDNA reappearance at M12 preceding liver and pulmonary metastases and patient 19 showed ctDNA clearance after initial treatment, followed by re-emergence before the appearance of lung metastases. In patients who were ctDNA-negative at baseline but later experienced progression, ctDNA became detectable either before or at the time of clinical decline (IDs 02 and 08). In both cases, ctDNA detection occurred despite the absence of measurable lesions at earlier time points and preceded a rapid deterioration of general condition. Persistent ctDNA at M0 and M4 was also associated with particularly aggressive disease courses. In patients 01 and 07, ctDNA remained detectable after surgery and throughout chemotherapy, and both developed early metastatic progression within the first year of follow-up. These patterns are reflected in Table 1, which reports ctDNA clearance, persistence, recurrence, and detection at progression across FIGO stage categories.

## 4. Discussion

In a population characterized by a high proportion of aggressive tumors and poor prognosis [14], the availability of an easily measurable prognostic biomarker is of particular importance. In this study, we monitored patients with high-grade EC by detecting somatic *TP53* mutations in plasma. Targeting *TP53*, a gene frequently mutated in these tumors, proved to be a relevant strategy. Because of the diversity of *TP53* variants, NGS was the most appropriate technical approach, and our method demonstrated sufficient sensitivity for ctDNA monitoring, as it allowed the detection of 16 of the 21 tumor mutations (73%). Mutations not detected in plasma occurred exclusively in patients diagnosed at FIGO stage I and/or those who did not relapse during follow-up, suggesting that the absence of detection was related to disease stage rather than analytical limitations. Previous studies have already shown that ctDNA detection is strongly associated with advanced disease [30,31]. In a context where patients are frequently diagnosed at a late stage, as confirmed by the survival data from our cohort (3-year RFS and OS rates of 41% and 44%, respectively), ctDNA analysis could complement pre-operative biopsy, particularly when the general condition of the patient is deteriorated and surgery is not feasible. This is illustrated by patient 16, for whom diagnosis was limited to curettage because of rapid clinical decline. Larger studies are needed to better evaluate the usefulness of ctDNA in patients who are not candidates for surgery. Beyond baseline prognostic stratification, our results highlight the clinical relevance of post-operative ctDNA monitoring. Persistent or recurrent ctDNA detection during follow-up was strongly associated with disease progression and poor clinical outcomes, whereas ctDNA clearance after treatment was associated with a more favorable course. In several patients, ctDNA dynamics closely mirrored response to therapy, supporting the concept of ctDNA as a marker of biological treatment response. Patients who achieved early ctDNA clearance after first-line chemotherapy or radiotherapy experienced delayed recurrence and longer survival, whereas persistent ctDNA was associated with early progression, suggesting underlying therapeutic resistance. Importantly, these findings indicate that ctDNA monitoring may provide additional value beyond outcome prediction by helping to identify patients at high risk of recurrence who may benefit from intensified post-operative surveillance. Patients with detectable or persistent ctDNA could be candidates for closer clinical follow-up, earlier imaging, or therapeutic adaptation, whereas patients with sustained ctDNA negativity might safely undergo standard surveillance schedules. This risk-adapted approach to follow-up could contribute to more personalized management strategies in high-grade endometrial cancer. The growing interest in serial ctDNA monitoring in oncology further supports our observations. The SERENA-6 trial, for instance, demonstrated that in HR+/HER2- breast cancer, early detection of *ESR1* resistance mutations in plasma allowed treatment adaptation months before radiological progression, thereby improving patient management [25,26]. In parallel, large precision-oncology programs such as PRISM and PRISM-PORTAL have shown that integrating broad ctDNA sequencing into clinical workflows enables real-time monitoring of tumor evolution and emerging resistance mechanisms [39]. Our study aligns with this strategy by demonstrating the feasibility of tracking *TP53* mutations in plasma in high-grade EC using a routine NGS assay. This approach represents a first step toward identifying mechanisms of recurrence and therapeutic resistance—such as replication-stress pathways linked to *CCNE1* amplification—and toward developing more personalized therapeutic strategies.

This study has limitations. Despite prospective recruitment, the sample size was small, and survival analyses were based on a limited number of events. Larger multicenter studies are required to validate these preliminary results. In addition, follow-up blood samples were collected every four months; although different intervals have been used in other studies [30,31,32], conclusions appear consistent across protocols. Defining the optimal monitoring interval will be essential if ctDNA is to be implemented clinically, particularly given the rapid progression of high-grade disease.

## 5. Conclusions

This study demonstrates that monitoring high-grade endometrial cancer through *TP53*-based ctDNA analysis is not only feasible but clinically meaningful. By showing that ctDNA detection mirrors disease stage, postoperative dynamics, and early progression, our work establishes liquid biopsy as a powerful tool for real-time assessment of tumor behavior in a population marked by an exceptionally high burden of aggressive disease. The ability to detect or clear ctDNA months before clinical deterioration underscores its potential as a prognostic and early-warning biomarker, paving the way toward integrating ctDNA into personalized surveillance strategies. Beyond *TP53* tracking, this study lays the groundwork for future approaches aimed at identifying molecular drivers of recurrence and therapeutic resistance—an effort aligned with emerging precision-oncology frameworks such as PRISM-PORTAL and trials like SERENA-6. Altogether, our findings highlight ctDNA as a transformative biomarker that could reshape the management of high-grade endometrial tumors, particularly in settings where aggressive phenotypes and delayed diagnosis remain major challenges.

## Figures and Tables

**Table 1:** Summary of ctDNA detection patterns.

Variable	FIGO I (*n* = 6)	FIGO > I (*n* = 15)	Total (*n* = 21)
Baseline ctDNA positive, *n* (%)	0	11	11
ctDNA clearance after first-line chemotherapy (M4)	0	3	3
Persistent ctDNA at M4 despite first-line chemotherapy	0	4	4
Delayed ctDNA clearance after radiotherapy (M8)	0	1	1
Early death before M4 (no post-op ctDNA evaluation)	0	3	3
No M4 sample, ctDNA positive at M8	0	1	1
ctDNA recurrence before radiologic progression	1	3	4
ctDNA detection concurrent with progression	0	1	1
ctDNA never detected during follow-up	4	1	5

## Data Availability

The datasets used and/or analysed during the current study are available from the corresponding author on reasonable request.
